# Enhanced bioenergetic cellular activity with metabolic switch to aerobic glycolysis in Keloid and Folliculitis Keloidalis Nuchae

**DOI:** 10.1007/s00403-024-03038-5

**Published:** 2024-06-15

**Authors:** Temwani Chalwa, Maribanyana Lebeko, Relebohile Matobole, Nonhlanhla P Khumalo, Ardeshir Bayat

**Affiliations:** MRC-SA Wound Healing and Keloid Research Unit, Division of Dermatology, Department of Medicine, Groote Schuur Hospital, University of Cape Town, Cape Town, South Africa

**Keywords:** Keloid, Folliculitis Keloidalis Nuchea, Cell bioenergetics, Metabolism, Glycolysis, Scarring

## Abstract

Keloid scars and folliculitis keloidalis nuchae (FKN) are benign fibroproliferative dermal lesions of unknown aetiology and ill-defined treatment, which typically present in genetically susceptible individuals. Their pathognomonic hallmarks include local aggressive invasive behaviour plus high recurrence post-therapy. In view of this, we investigated proliferative and key parameters of bioenergetic cellular characteristics of site-specific keloid-derived fibroblasts (intra(centre)- and peri(margin)-lesional) and FKN compared to normal skin and normal flat non-hypertrophic scar fibroblasts as negative controls.

The results showed statistically significant (*P* < 0.01) and variable growth dynamics with increased proliferation and migration in keloid fibroblasts, while FKN fibroblasts showed a significant (*P* < 0.001) increase in proliferation but similar migration profile to controls. A statistically significant metabolic switch towards aerobic glycolysis in the fibroblasts from the disease conditions was noted. Furthermore, an increase in basal glycolysis with a concomitant increase in the cellular maximum glycolytic capacity was also demonstrated in perilesional keloid and FKN fibroblasts (*P* < 0.05). Mitochondrial function parameters showed increased oxidative phosphorylation in the disease conditions (*P* < 0.05) indicating functional mitochondria. These findings further suggest that Keloids and FKN demonstrate a switch to a metabolic phenotype of aerobic glycolysis. Increased glycolytic flux inhibition is a potential mechanistic basis for future therapy.

**Supplementary Information:**

The online version contains supplementary material available at 10.1007/s00403-024-03038-5.

## Introduction

Pathological scarring occurs commonly when the normal wound healing response is derailed and in the susceptible individual, this may lead to the formation of abnormal excessive dermal scarring conditions such as keloids [1] and/or folliculitis keloidalis nuchae (FKN) [2]. This occurs when the delicate balance between the extracellular matrix (ECM) components and the degradation of these components becomes perturbed [3]. Keloids and FKN have a similar clinical presentation although FKN is localised to one anatomical site (the scalp) whereas keloids can occur at any site. Moreover, the susceptible individual who gets a keloid or FKN may not necessarily harbour both lesions.

Keloids are common benign fibroproliferative cutaneous exophytic outgrowths that occur post wounding or trauma to the skin [4]. Unique to humans, familial inheritance is strong, and their occurrence is higher in African, Hispanic and Asian descent. They exhibit a poor clinical outcome with high rates of recurrence post therapy (1, 5–6).

FKN, often incorrectly referred to as acne keloidalis (AKN), is a dermal fibrotic condition that is localised to the nuchal region of the scalp and features chronic inflammation of the hair follicles with papule formation. The initially formed papules with exiting hair tend to merge to form prominent firm keloid-like nodules and plaques eventually leading to scarring alopecia [2, 7]. Lesions start on the nuchae and may extend to the rest of the scalp [8, 9]. The exact aetiology remains unknown, however, in-grown hairs have been suggested as a trigger because of high prevalence of FKN in populations with curly hair who have close-shave haircuts [10–12].

Metabolism is fast becoming an area of focus in research in fibrotic disorders. In lung fibrosis, defective fatty acid metabolism has been shown to promote disease [13]. Defective metabolism is also a factor in the pathogenesis of kidney fibrosis [14]. Moreover, a reprogramming of energetic metabolism has been reported in chronic diseases [15, 16].

**Fig. 1:**
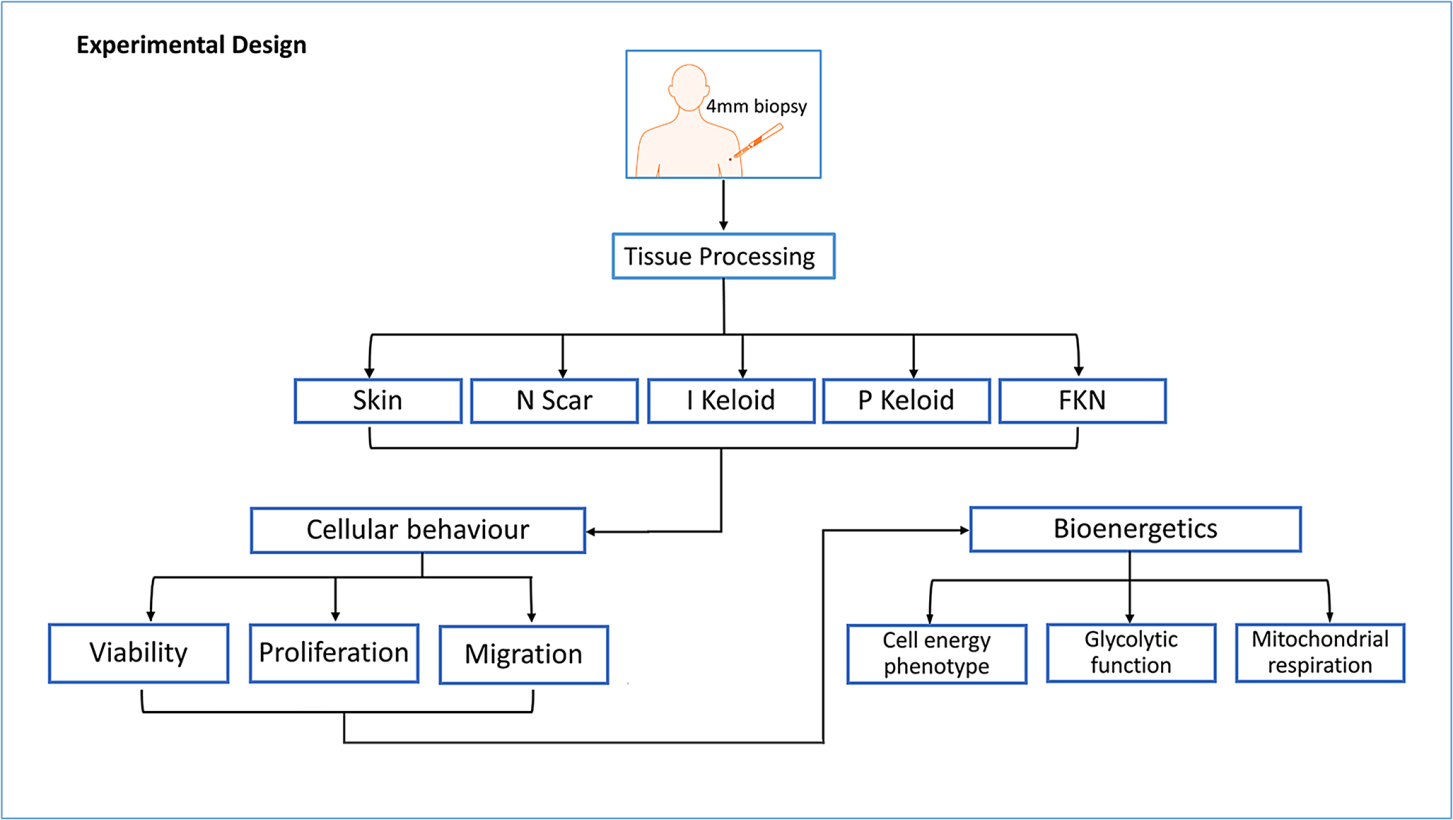
Illustration of the experimental design and methodology of the study. Various cellular behavioural components of site-specific keloid and FKN scars were compared against normal skin and normal scar controls. Subsequently, differences in the bioenergetics of these cells from the different lesions were established. Skin - normal non-scarred skin; N Scar - normal flat non-hypertrophic scar; I Keloid - intralesional keloid; P Keloid - perilesional keloid, FKN - folliculitis keloidalis nuchae

A hallmark of the metabolism of cancer cells is the capacity to obtain and utilise nutrients needed for the maintenance of the cell’s viability plus increasing the biomass. This leads to changes in cellular differentiation and tumour microenvironment. Known cancer-associated metabolic reprogramming includes the use of the glycolysis/Krebs cycle often exhibiting the well-defined Warburg effect of aerobic glycolysis. The glycolysis/Krebs cycle intermediates are utilised for biosynthesis as well as NADPH production. In addition to changes in the uptake of glucose and amino acids as well as changes in the metabolic interaction with the tumour microenvironment, gene regulation is also altered [17–19].

Some studies have noted a disruption in the metabolism of Keloids in recent years [20–27] while we could not find any cellular or molecular studies on FKN. Evidently, more studies need to be performed to understand abnormal pathological scarring, with respect to metabolic perturbations.

Hence, the aim here was to investigate cellular bioenergetics in two clinically distinct yet metabolically active dermal fibrotic disorders; Keloid and FKN fibroblasts. Furthermore, we compared our findings to normal (normal skin and normal flat non-hypertrophic scarring) in order to establish whether these deviations from normal may be linked to or influence changes in bioenergetics in these fibroblast cells (Table 1) (Fig. 1). The findings here may elucidate the role of metabolic reprogramming at specific lesional sites.

**Table 1:** Information of study patients and healthy volunteers

Group	Participant age	Age of lesion (years)	Site	Ethnicity	Sex
**Normal skin**	32	0	left arm	Black	M
	35	0	left arm	Black	M
	30	0	left arm	Black	M
	40	0	left arm	Black	M
	30	0	left arm	Black	M
**Normal scar**	52	40	left leg	Black	F
	32	21	right arm	Black	M
	22	14	abdomen	Colored	F
**Keloid**	36	3	nape of neck	Black	M
		25–27	upper left arm		
	31	7	right earlobe	Black	M
	38	15	upper right arm	Black	F
		16	right earlobe		
**FKN**	30	6	scalp	Black	M
	27	8	scalp	Black	M
	30	17	scalp	Black	M

## Results

### Establishment of fibroblast cell growth dynamics in different conditions

In order to begin to characterise the differences in cellular behaviour from the 5 conditions (intra- and peri-lesional keloid, FKN, normal skin and normal scarring), first the proliferating ability of the cells were assessed. This was done to establish possible differences in their growth patterns. One of the clinically defining characteristics of keloids and FKN is their increased rate of matrix deposition. By observing the proliferation of the cells, inference could be made about whether it is a result of increased numbers of fibroblasts or whether similar numbers of fibroblasts are responsible for the matrix deposition.

**Fig. 2:**
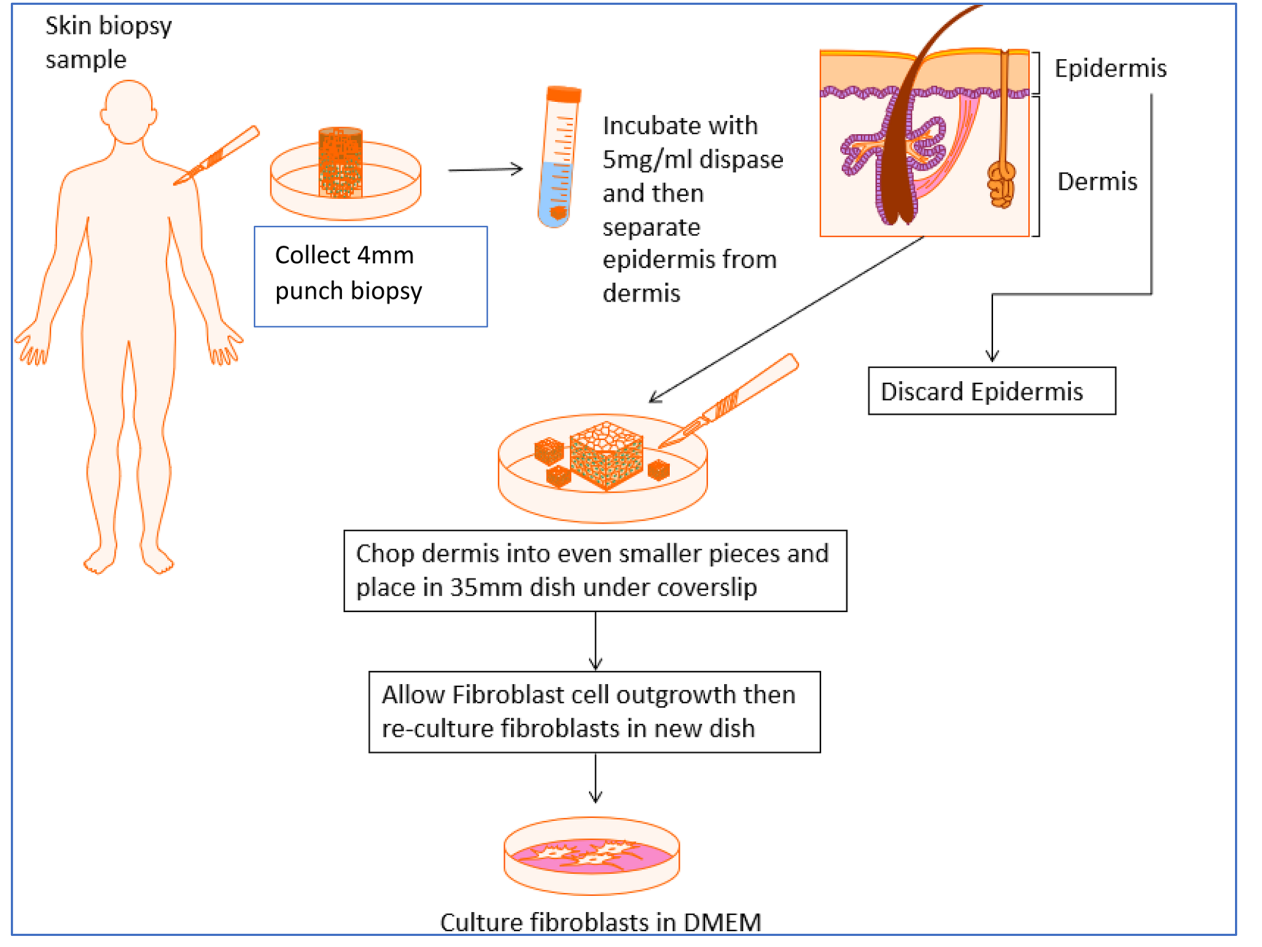
Summary of isolation and cell culture protocol of primary human dermal fibroblast cells. Figure created with Motifolio Toolkit (Motifolio Inc, Ellicott City, MD)

**Fig. 3:**
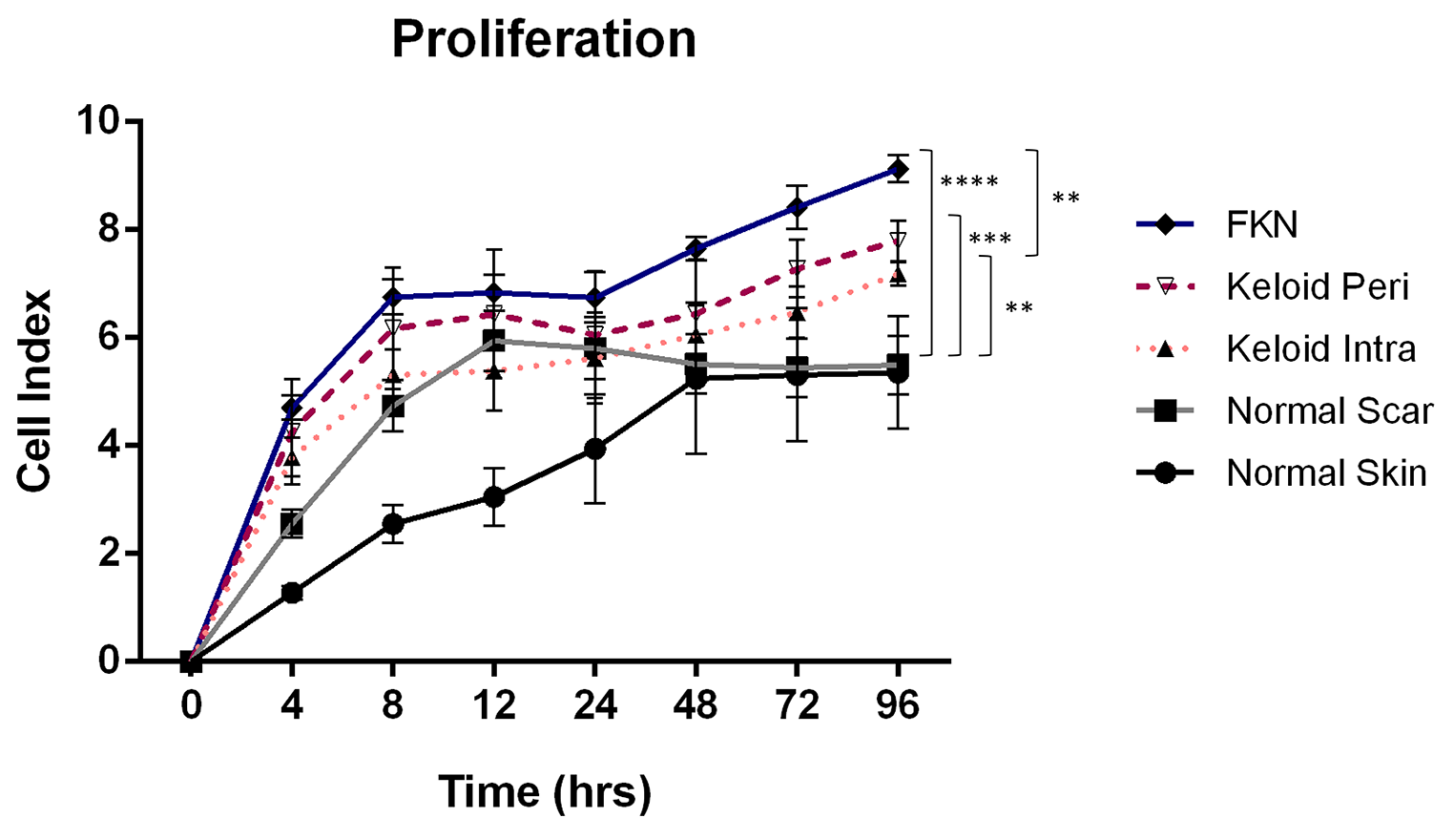
Inherent fibroblast cellular proliferation profiles measured by Real Time Cell Analyzer (RTCA). An xCELLigence RTCA instrument was used to measure the proliferation of the cells by growing cells in 10% FBS growth factor at a density of 1 × 10^4^ cells per well of the RTCA plate. Growth curves were conducted over a 4-day period. Results are representative of data from 3 independent biological experiments with 2 replicates. Significance was set as *, *P* < 0.05; **, *P* < 0.01; ***, *P* < 0.001; ****, *P* < 0.0001; Two-way ANOVA with Tukey HSD post-hoc test

The inherent proliferation of the cells was measured in real time using an xCELLigence Real Time Cell Analyzer (RTCA). Initially, 1 × 10^4^ cells were plated per well of the RTCA plate and growth curves were constructed from these measurements over a 4-day period. As shown in Fig. 3 there were significant differences in the growth dynamics of the cells from the 5 different conditions. By the 72 h time point there was a marked increase in proliferation in the disease conditions relative to the controls as indicated by the cell index. After the 4-day period, the NS (normal skin) and NSc (normal scar) fibroblasts had mean cell indices of 5.36 ± 1.04 and 5.50 ± 0.54 respectively, whereas the keloid disease conditions had mean cell indices of 7.19 ± 0.21 and 7.79 ± 0.38 for KI (intralesional keloid) and KP (perilesional keloid) fibroblasts respectively. FKN had the highest mean cell index at 9.13 ± 0.25. Keloids and FKN had significantly higher proliferation than the controls, and it was further noted that FKN cells had a significantly higher proliferation than KI cells but not KP cells. There was no significant difference between the proliferation rates of the NS and NSc controls, and this was also noted between KI and KP as well even though KP was higher than KI.

Keloid fibroblasts show increase in migration ability while FKN fibroblasts have similar migration profile compared to controls.

After the proliferating ability of the fibroblasts in the different conditions was assessed, the migratory capacity was the next characteristic to be determined using scratch motility assays.

**Fig. 4:**
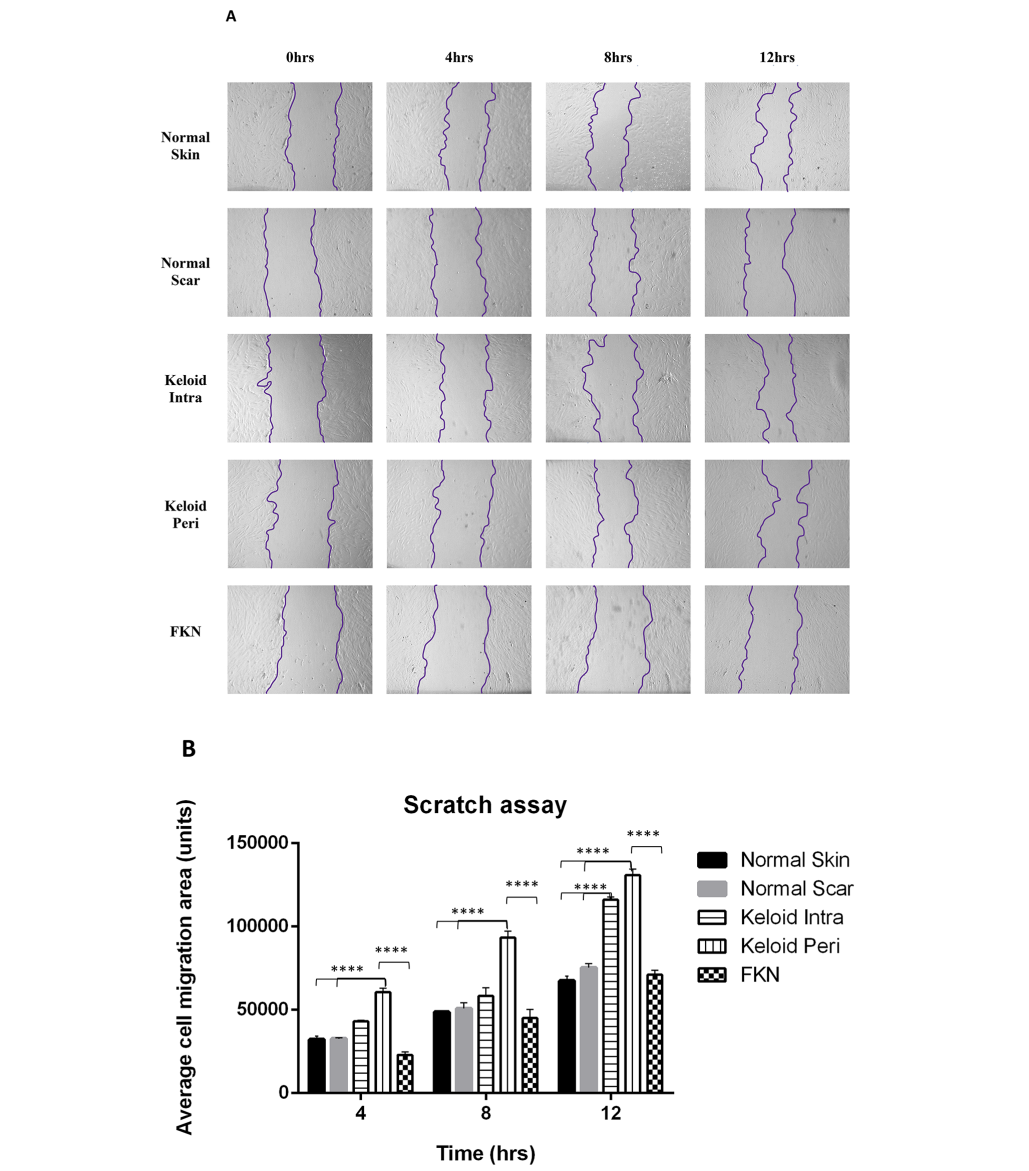
Scratch motility assay determination of the migratory ability of various disease and control dermal fibroblasts. (**A**) 2D motility assay was used to establish the migration. A linear wound was made by scratching through the monolayer with a pipette tip after cells were confluent. Wound closure was measured over a 12 h period. (**B**) Quantification of average cell migration area. Keloids displayed increased migration. Results are representative of data from 3 independent biological experiments with 3 replicates. Significance was set as *, *P* < 0.05; **, *P* < 0.01; ***, *P* < 0.001; ****, *P* < 0.0001; Two-way ANOVA with Tukey HSD post-hoc test

The results (Fig. 4a, b) show that the average area migrated was significantly higher in both KI and KP fibroblasts at the 4 h and 12 h mark in comparison to the NS and NSc controls which had similar migration profiles. At the 8 h mark only KP fibroblasts were significantly different to the controls.

Interestingly, FKN fibroblasts showed no significant difference in migration to the controls at all the time points, even though it followed a similar trend of an increase in migration at each time point.

It is evident that both Keloid fibroblasts exhibit high migration rates, yet FKN fibroblasts exhibit normal migration capacity compared to other conditions and normal skin fibroblasts.

### Keloid and FKN fibroblasts have different energy phenotype profiles

Due to the differences in cellular behaviour that were observed, bioenergetic characterisation experiments were performed in real time using the Agilent extracellular flux analyser. First, a cell energy phenotype experiment was carried out to see if there were basic differences in the energy phenotypes of the various disease conditions relative to the controls. The Oxygen Consumption Rate (OCR) which acts as a proxy to mitochondrial respiration and Extracellular Acidification Rate (ECAR) which acts as a proxy to glycolysis, were measured under baseline and stressed conditions. This gives an indication of the metabolic potential of the cells.

**Fig. 5:**
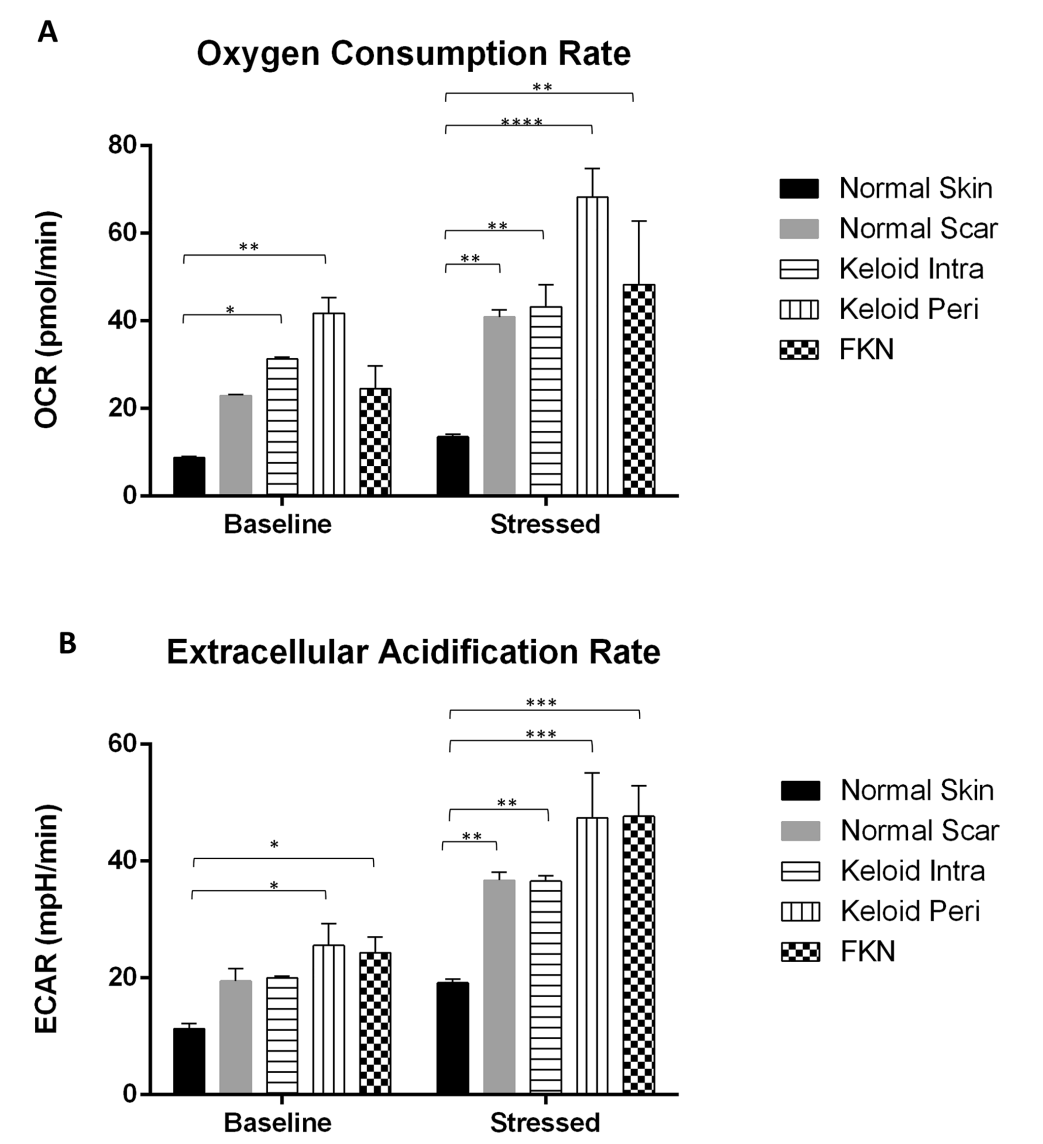
General cell energy phenotype assays including measurement of the Extracellular Acidification Rate (ECAR) and Oxygen Consumption Rate (OCR) in various disease and control dermal fibroblasts. Results of Cell Energy Phenotype XF Flux analyser test for both (**A**) OCR (pmol/min) and (**B**) ECAR (mpH/min) simultaneously. Keloid and FKN fibroblasts showed increased metabolic potential. Results are representative of data from 4 independent biological experiments with 3 replicates. Significance was set as *, *P* < 0.05; **, *P* < 0.05; **, *P* < 0.01; ***, *P* < 0.001; ****, *P* < 0.0001; Two-way ANOVA with Tukey HSD post-hoc test

There was a significant difference in the baseline OCR between the NS control and both the KI and KP fibroblast groups (Fig. 5a). The baseline OCR of the NS fibroblasts was 8.84 ± 0.22 pmol/min while KI was 31.37 ± 0.33pmol/min and KP was 41.78 ± 3.51pmol/min. The difference in baseline OCR was insignificant in the other groups. Under stressed conditions using an Oligomycin and Carbonyl cyanide-4 (trifluoromethoxy) phenylhydrazone (FCCP) mixture, the OCR increased and there was more than a 3-fold difference. The increment was to 13.56 ± 0.59pmol/min in NS fibroblasts, while it increased to 43.24 ± 5.00pmol/min in KI and 68.21 ± 6.63pmol/min in KP. There was also a significant difference between NS and FKN which increased to 48.28 ± 14.57pmol/min.

The baseline ECAR (Fig. 5b) shows a significant difference between NS 11.33 ± 0.84mpH/min and KP and FKN which are 25.56 ± 3.75mpH/min and 24.31 ± 2.68mpH/min respectively. After the Oligomycin and FCCP stressor mixture, there is a significant difference between NS and the other groups. The NS group ECAR increased to 19.19 ± 0.62mpH/min, while NSc 36.73 ± 1.39mpH/min, KI 36.57 ± 0.96mpH/min, KP 47.38 ± 7.72mpH/min and 47.68 ± 5.24mpH/min for FKN. Therefore, Keloid (more so KP) and FKN Fibroblasts have different energy phenotype profiles.

### Keloid and FKN fibroblasts exhibit amplified glycolysis

**Fig. 6:**
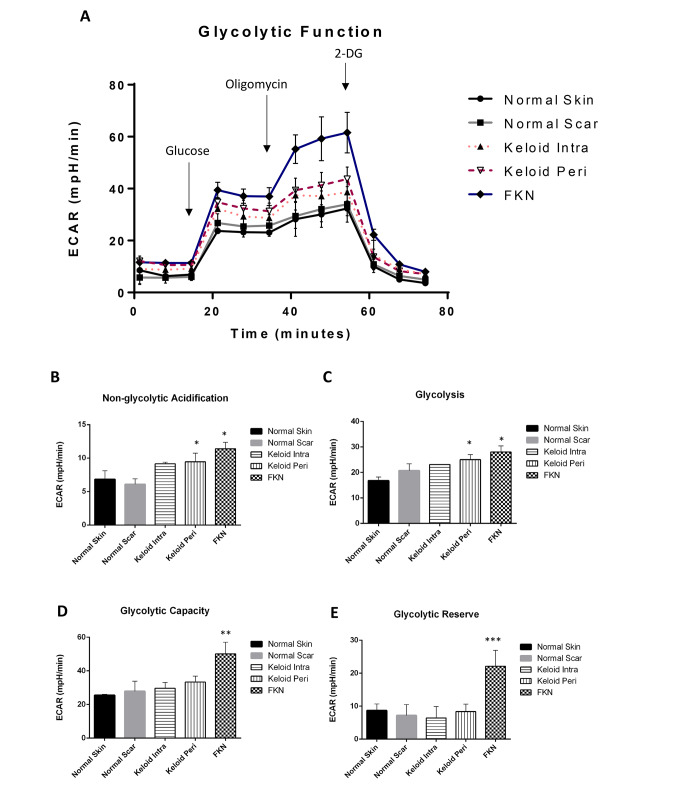
Bioenergetics parameters related to glycolysis measured by Extracellular Acidification Rate (ECAR) in various disease and control dermal fibroblasts. (**A**) Profiles of Glycolysis Stress XF Flux analyser measurements of ECAR (mpH/min) in disease fibroblasts relative to controls. Arrows indicate injection of glucose and the specific stressors into the media. (**B**-**E**) Values of the different parameters of mitochondrial respiration. Keloid and FKN fibroblasts exhibit augmented glycolysis. Results are representative of data from 4 independent biological experiments with 3 replicates. Significance was set as *, *P* < 0.05; **, *P* < 0.01; ***, *P* < 0.001; Two-way ANOVA with Tukey HSD post-hoc test

Owing to the significant difference between controls and the disease conditions in basic energy phenotype experiments, the Agilent XF flux analyser Glycolysis Stress Test was used to further analyse the ECAR. This was done to determine whether the pathology of keloids involved the disruption of glycolytic pathways, hence affecting glycolytic function. Cells were seeded and first observed under baseline conditions with subsequent injections of glucose, followed by Oligomycin and lastly 2-DG (Fig. 6a).

ECAR measured under baseline conditions is considered non-glycolytic acidification. It was significantly higher in KP and FKN, 9.45 ± 1.28mpH/min and 11.40 ± 0.95mpH/min respectively, in comparison to 6.85 ± 1.25mpH/min and 6.1 ± 0.80mp/min for NS and NSc respectively (Fig. 6b). Activation of glycolysis occurs after the addition of glucose, and this is considered basal glycolysis. There was a significant difference between NS 16.80 ± 1.40mpH/min and KP and FKN, 25.00 ± 1.97mpH/min and 28.03 ± 2.34mpH/min respectively (Fig. 6c). After the Oligomycin injection to make glycolytic function maximal hence giving the overall glycolytic capacity, there was also a significant difference between NS 25.5 ± 0.60mpH/min and KP and FKN, 33.35 ± 3.55mpH/min and 50.17 ± 6.91mpH/min respectively (Fig. 6d). The glycolytic reserve which is the difference between the maximal and basal glycolysis was similar in all the groups except the FKN disease group where the glycolytic reserve was more than 2-fold higher than the other conditions (Fig. 6e). When 2-DG is injected, ECAR drops significantly to non-glycolytic levels because it acts as a non-competitive inhibitor of the glycolytic pathway. Evidently, Keloid (more so KP) and FKN fibroblasts exhibit amplified glycolysis relative to the controls.

### Keloid and FKN fibroblasts have functional mitochondria

**Fig. 7:**
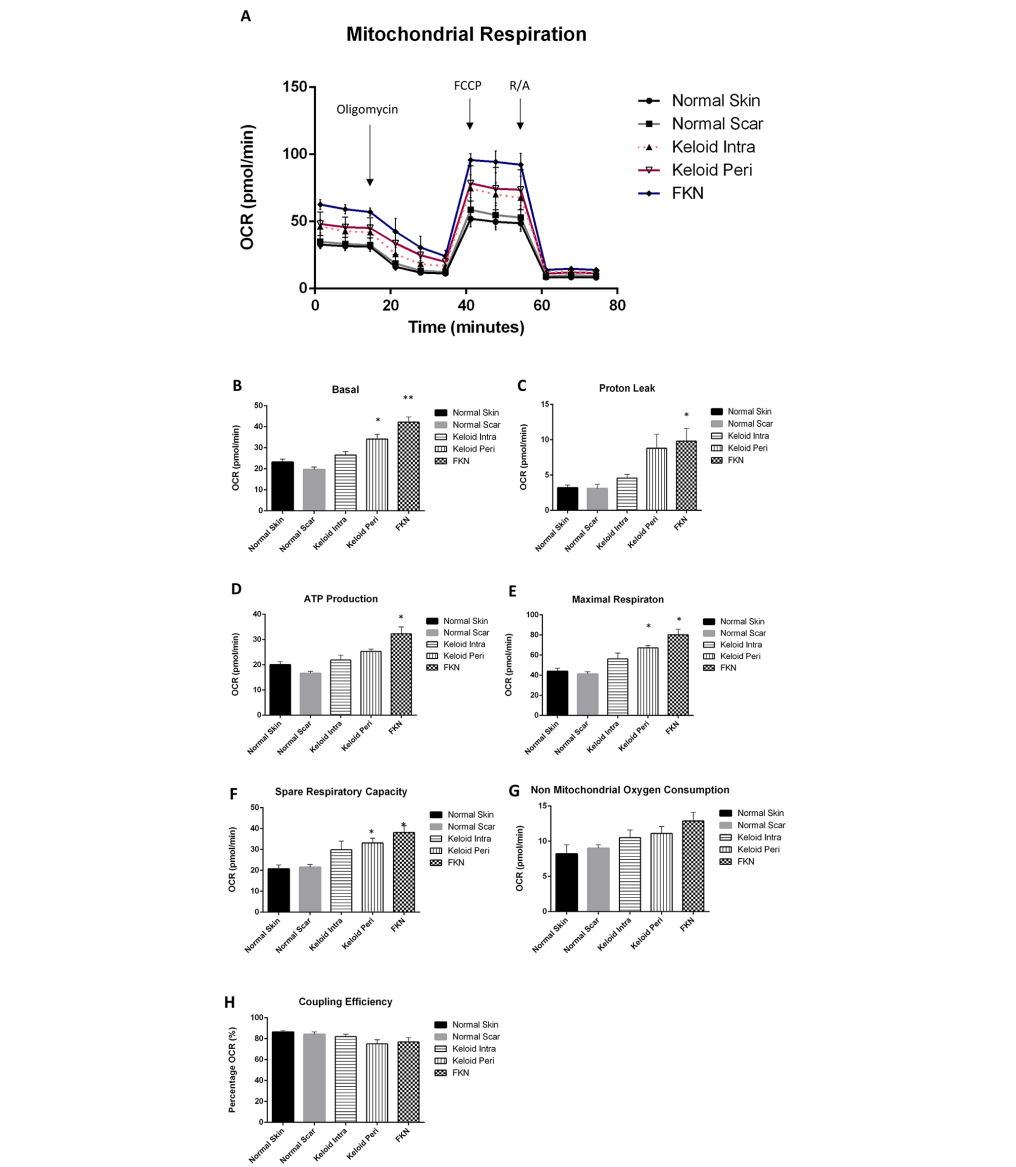
Bioenergetics parameters related to respiration in mitochondria measured by Oxygen Consumption Rate (OCR) in various disease and control dermal fibroblasts. (**A**) Profiles of Mito Stress XF Flux analyser measurements of OCR in disease fibroblasts relative to controls. Arrows indicate injection of the specific stressors into the media. (**B**-**H**) Values of the different parameters of mitochondrial respiration. Keloid and FKN fibroblasts have functional mitochondria capable of mitochondrial respiration. Results are representative of data from 3 independent experiments with 3 replicates. Significance was set as *, *P* < 0.05; **, *P* < 0.01; ***, *P* < 0.001; Two-way ANOVA with Tukey HSD post-hoc test

Following the above results showing a shift towards glycolysis in Keloid and FKN fibroblasts, functional measurement of mitochondrial parameters was carried out to determine if the mitochondria in these fibroblasts were still functional. To do this, the Agilent XF Cell Mito Stress Test which looks at the OCR was used. Cells were seeded and first observed under baseline conditions with subsequent injections of Oligomycin, followed by FCCP uncoupling agent and lastly a Rotenone and Antimycin A inhibitor cocktail (Fig. 7a).

The results showed a significant increase in OCR measured under basal conditions in disease conditions relative to the controls. The basal respiration was the lowest in NSc at 19.7 ± 1.1pmol/min and was at 23 ± 1.4pmol/min in NS fibroblast cells. The Keloid fibroblasts had basal respiration rates of 26.5 ± 1.6pmol/min and 34.1 ± 2.3pmol/min for KI and KP respectively, while FKN had the highest basal respiration rate at 42.2 ± 2.5pmol/min (Fig. 7b).

Following injection of the Oligomycin to determine ATP-linked respiration and proton leak, there was a significant difference in ATP-linked respiration between NSc 16.6 ± 0.8pmol/min and the disease conditions at 25.3 ± 0.9pmol/min and 32.3 ± 2.7pmol/min for KP and FKN respectively. There was no statistically significant difference between NS fibroblasts (20.0 ± 1.2pmol/min) and Keloids but a significant difference was recorded between NS fibroblasts and FKN. The proton leak was only significantly different between FKN at 9.8 ± 1.8pmol/min and the controls at 3.2 ± 0.4pmol/min and 3.1 ± 0.6pmol/min for NS and NSc respectively (Fig. 7c).

Maximal respiration is established after injection of FCCP and a significant difference between the disease and control conditions was recorded. NS and NSc fibroblasts had maximal respiration rates of 43.9 ± 3.1pmol/min and 41.2 ± 2.3pmol/min respectively. In comparison, KI was at 56.3 ± 5.7pmol/min, KP was at 67.3 ± 2.2pmol/min and FKN was almost double the controls at 80 ± 5.5pmol/min (Fig. 7e). This gave the disease conditions a much higher spare respiratory capacity than the controls. The spare respiratory capacity is the difference between the maximal respiration and the ATP-linked respiration (Fig. 7f).

After the Rotenone and Antimycin inhibitor cocktail injection, there is a rapid decrease in mitochondrial respiration as the mitochondrial processes are shut down. This enables the quantification of the non-mitochondrial respiration which is a result of processes outside the mitochondria (Fig. 7g). The non-mitochondrial oxygen consumption was higher in the disease conditions in comparison to the controls although this difference was not statistically significant. The reverse was true for the coupling efficiency (Fig. 7g, h). Thus, Keloid and FKN fibroblasts also have functional Mitochondria.

## Discussion

To determine differences, first the proliferating ability of disease versus control cells were investigated using Real Time Cell Analysis. Growth curves performed over the 4-day period showed that both intralesional (KI) and perilesional keloid fibroblasts (KP) displayed an increase in proliferation relative to normal scar (NSc) and normal skin (NS) controls with KP fibroblasts showing a higher rate than KI. The FKN fibroblasts showed an even higher increase in proliferation than both Keloid fibroblast subtypes (Fig. 3).

The difference in proliferation between KI and KP fibroblasts is supported by a study that showed differential gene expression between margin and centre of keloids [28]. Clinically, margin is where keloid mass is actively growing and invading normal surrounding skin, and this contrasts with the centre which exhibits less growth and reduced activity. This suggests that fibroblasts from the margin are in a more activated/energetically upregulated state in comparison to the centre. Due to lack of research on the proliferation dynamics of fibroblasts in FKN, no comparison can be made to other studies.

Interestingly, NSc fibroblasts showed a rapid increase in cell index which started to plateau early at the 48 h mark (Fig. 3). At this point they had undergone contact inhibition and stopped proliferating [29]. This rapid increase in cell index could be due to the larger size of these fibroblasts which are morphologically similar to a more senescent phenotype.

In vitro migratory ability of the fibroblasts from the different skin conditions showed a significant increase in the migration of both KI and KP fibroblasts relative to controls. However, FKN exhibited a similar migration profile to controls.

This is in agreement with studies where significant increase in migration of Keloid fibroblasts were reported [30, 31]. This increase in migration likely attributed to upregulation in focal adhesion kinase demonstrated in mechano-sensing during fibroblast migration [32] or an upregulation of matrix metalloproteinases [31]. FKN fibroblasts followed a similar trend to the controls. This could imply that the cells may be less invasive in comparison to Keloid fibroblasts. This may explain the difference in post-excision recurrence rate, which is inconsistent in FKN [33] and up to 100% in keloids post-excision alone [34]. However, as with the proliferation assays, there have been no published studies on the migration of FKN fibroblasts.

From the differences in the cellular phenotype observed after the proliferation and migration assays, a Cell Energy Phenotype test was performed. This test helps establish the metabolic potentials of the cells for Oxygen Consumption Rate (OCR) and Extracellular Acidification Rate (ECAR).

This showed a significant increase in baseline and stressed levels and of both OCR and ECAR, for the Keloid fibroblasts relative to control. This indicates overall increased metabolic potential in keloid fibroblasts. FKN also exhibited a significant increase relative to the NS fibroblasts in all but the baseline OCR. The OCR was higher in NS fibroblasts under stressed conditions, therefore the data suggests that FKN fibroblasts have increased mitochondrial activity but may have an impairment to their overall mitochondrial metabolic potential as OCR was low.

Unexpectedly, NSc fibroblasts also exhibited increased levels of both OCR and ECAR under stressed conditions to similar levels as KI fibroblasts. This suggests that NSc fibroblasts undergo some similar changes to their bioenergetic pathways even though the changes are not as extreme as seen in Keloids and FKN.

To further determine if glycolytic dysregulation and its potential effects on glycolytic flux is a factor in the transformed energy phenotype, the ECAR was measured via the Glycolysis Stress test (Fig. 6a). ECAR is used as a substitute to measure lactate export which is a product of glycolysis and is a proxy to monitoring glycolytic flux.

Baseline ECAR measurements were first taken indicating non-glycolytic acidification which was significantly higher in Keloid (more in KP) and FKN fibroblasts relative to controls (Fig. 6b). This finding showed that in the absence of glucose, the fibroblasts in these disease conditions produce more acid than controls. When glucose was injected, glycolysis is activated, and readouts are at basal glycolytic levels (Fig. 6c). The fibroblasts from the disease conditions have a higher increase relative to the controls showing that there are higher glycolysis basal levels in Keloid (more in KP) and FKN fibroblasts than controls. After addition of Oligomycin, mitochondrial respiration is shut down as it is an ATP synthase inhibitor. This creates an increase in glycolytic flux as the other energy source is blocked resulting in the maximum glycolytic capacity. The disease fibroblasts showed a greater maximum glycolytic capacity than controls, with FKN fibroblasts being significantly higher than keloid fibroblasts. Finally, 2-DG gets injected into the medium and glycolysis is blocked (Fig. 6a) as 2-DG is a competitive glucose analogue that binds the hexokinase at the beginning of glycolysis. These results are supported by the recent studies that also looked at Keloid cells and their metabolic reprogramming. An increase in glycolysis and the other glycolytic parameters were also recorded in these studies [23, 27]. Glycolytic inhibitors such as 3-Bromopyruvate which inhibits hexokinase activity hence abolishing ATP production, or Oxamic acid which inhibits lactate dehydrogenase, may be used [35].

Due to the establishment of glycolytic dysregulation as a factor in the transformed energy phenotype, the study then went on to analyse mitochondria and associated parameters of mitochondrial respiration. The OCR was measured via the Mito Stress test (Fig. 7a). OCR is used as a proxy to monitor mitochondrial respiration.

Baseline OCR measurements were first taken indicating basal respiration was significantly higher in KP and FKN fibroblasts relative to controls (Fig. 7b). When Oligomycin gets injected, this leads to inhibition of ATP synthase and as a result creates a drop in OCR or mitochondrial respiration. This drop is indicative of ATP production in the mitochondria (Fig. 7d) and residual OCR is considered proton leak (Fig. 7c). ATP production was found to be higher in KP and FKN fibroblasts and proton leak was also significantly higher in these disease fibroblasts. This supports suspicion of impairment to their overall mitochondrial metabolic potential alluded to above. Interestingly, KP and FKN fibroblasts have the highest rate of proton leak, but at the same time they exhibit the highest ATP production as well as maximal respiration. This phenomenon could be due to increased mitochondrial numbers or size in these disease fibroblasts. This would account for the increased ATP production and maximal respiration exhibited. After addition of FCCP, this uncoupling agent collapses the mitochondrial membrane potential, and electrons flow unimpeded through the electron transport chain. This results in maximal oxygen to complex IV of the electron transport chain hence maximal OCR (Fig. 7e) to give the spare respiratory capacity (Fig. 7f). This spare capacity signifies the capabilities of the cell to withstand increased demand for energy and is higher in the disease conditions relative to the controls. The final injection is Rotenone and Antimycin A which block complexes I and III respectively allowing the determination of non-mitochondrial respiration [36].

Keloid and FKN fibroblasts had significantly higher spare respiratory capacities. This signifies that even though these fibroblasts have functional mitochondria with even greater capacity for energy demand than the controls, they utilise glycolysis. This switching to aerobic glycolysis is known as the Warburg effect and has been shown to be a hallmark of cancer cells [37]. It is a less efficient energy production mechanism and as such it’s exact function has come into debate [38]. One function of this effect is that cells utilise it as an adaptive mechanism to support biosynthesis of molecules needed to aid the process of increased proliferation. Glucose gets used as a carbon source during anabolism via the pathways that branch off of the main glycolytic pathway [39, 40]. Oxidative stress due to the hypoxic environment of keloid fibroblasts has been suggested as a reason for the Warburg effect, as a significant upregulation in the generation of ROS in keloid fibroblasts was noted [41]. These cells may switch to glycolysis as a defence mechanism against oxidative damage.

FKN cells appear to be more competent in both glycolysis and mitochondrial respiration as shown by elevated levels of both OCR and ECAR after stressors. This may explain their increased proliferation capacity as they utilise both energy metabolism pathways for their rapid multiplication solely to increase biomass and not to increase migration. Additional, in vitro experiments on FKN are required to further explain this phenomenon.

Altogether this study begins to provide insight into bioenergetics of normal scars and aberrant scarring diseases like keloid and FKN. Further insight is offered into the bioenergetics of the centre and margin of the Keloid. There was an overall increase in the growth dynamics of disease fibroblasts compared to normal skin (NS) and normal scar (NSc) fibroblasts.

Due to the heterogeneity of Keloid and FKN lesions, future studies are needed which should incorporate more participants with lesions from multiple sites, and intralesional differences should also be considered.

## Electronic supplementary material

Below is the link to the electronic supplementary material.

Supplementary Material 1

## Data Availability

No datasets were generated or analysed during the current study.
