# Supplementary material for: Enhanced bioenergetic cellular activity with metabolic switch to aerobic glycolysis in Keloid and Folliculitis Keloidalis Nuchae
Source: Arch Dermatol Res. 2024 Jun 15;316(7):412. doi: 10.1007/s00403-024-03038-5 (PMC11180017; doi:10.1007/s00403-024-03038-5)
Supplement: Supplementary file 1 — Supplementary Material 1 [file 403_2024_3038_MOESM1_ESM.docx]

**MATERIALS AND METHODS**

1. **Sample collection**

Ethics approval for the study was granted by Groote Schuur Hospital and University of Cape Town’s Human Research Ethics Committee. HREC REF No. 287/2018. Participants of this study consisted of consenting adult patients that were scheduled for Keloid or FKN surgical excision at the Outpatients Department of Plastic and Reconstructive Surgery unit of Groote Schuur Hospital. Additionally, consenting adult volunteers were used for normal skin and normal flat non-hypertrophic scar controls. This study was part of a larger study looking at different aspects of Keloids and FKN. For this portion of the study, 4mm punch biopsies were taken from all the participants for the culture of dermal skin Fibroblasts (Table 1). For the Keloid disease, both perilesional and intralesional samples were collected to make comparative analysis between the two sites as reports have shown differential gene expression between the margin and the centre of the keloid^28^. Firm puritus regions were selected for FKN to avoid regions possibly undergoing active infection and inflammation. The punch biopsies were collected in Dulbecco’s Modified Eagle’s Medium (DMEM) (Life Technologies, SA).

1. **Isolation of primary skin cells from tissue**

Fibroblasts from the 2 disease conditions as well as the normal skin and normal flat non-hypertrophic scar controls were cultured from the 4mm skin punch biopsies (fig. 2). The skin tissue specimen first went thorough removal of the epidermis to separate melanocytes, keratinocytes and other cells found in the epidermis from the dermal tissue of interest. This was done through overnight incubation with 5mg/ml dispase at 4°C followed by peeling off the epidermis using forceps (B & M Scientifics, SA). Thereafter the remaining dermal tissue was cut up into small fragments using a scalpel blade (B & M Scientifics, SA) and underwent preparation for culture, which included washing with PBS and antibiotic treatment. The dermal tissue was then placed in 2 ml of culture medium comprising DMEM, 10% foetal bovine serum (Life Technologies, SA) with 1% penicillin-streptomycin antibiotic (Life Technologies, SA). The cultures were then maintained in a humidified incubator (WhiteSci, SA) at 37 °C with a 5% carbon dioxide atmosphere to allow growth of fibroblast cells. After sufficient outgrowth of these cells, they were sub-cultured for further experiments. Only cells below passage 5 were used in all of the experiments of this article to preserve the inherent characteristics of the different conditions. All experiments were carried out with representative paired samples for the different experimental groups.

1. **Viability and Proliferation**

To determine the cell growth dynamics in the different conditions, an xCELLigence Real Time Cell Analysis (RTCA) Instrument (ACEA Biosciences Inc., USA) was used. This instrument utilizes electrical impedance in specialized 8-well E-plates containing gold microelectrodes imprinted in each individual well. These microelectrodes are used to monitor the adhesion of cells, and this is done by recording the electrical impedance that the cells cause in real time. Cells attaching to the microelectrodes cause a change in electronic readings which are output and plotted as cell index (CI) values, giving a precise indication of the viability and number of cells. Cells were then seeded at a density of 2 x 10^4^ cells per well and the experiment was carried out for 4 days.

1. **Fibroblast cellular migration**

A 2-dimensional in vitro scratch motility assay was used as a wound healing model to investigate the migration profile of cells. Following culture of the cells to confluence in a 12-well culture plate, a scratch was made using a 10μL pipette tip through the monolayer. This was made to simulate a wound and a ruler was used to guarantee that consistent “wounds” across the stripped area were made. The medium was then replaced with 2ml of fresh medium to get rid of floating cells and debris that occurred from the scratching process. Thereafter, to prevent further proliferation of the cells a mitogen inhibitor, Mitomycin C (Sigma, USA), was then added at a final concentration of 0.1μg/μl. Across each scratch line, three marks were made on the underside of the dish to create reference points for taking images with an inverted light phase contrast microscope (EVOS, life Technologies, SA). Three images that spanned the length of the scratch that was made in each well of the tissue culture plate were then captured at the time of the scratch (denoted t = 0h). Subsequently, wound closure was monitored by the capture of images in a similar fashion at specified time points. For each of the three images captured at each time point, the area that was denuded was measured using Image J software (National Institutes of Health, USA) and the rate of migration was subsequently graphed:

1. **Extracellular Flux analysis**

Comparisons between the metabolic phenotypes of the cells from the disease conditions as well as the 2 control groups were then carried out. The Seahorse XFe96 Flux Analyser (Agilent Technologies, USA) which works based on the extracellular acidification rate (ECAR) and Oxygen consumption rate (OCR), was used for this purpose. The flux analyser utilises a sensor cartridge that monitors these two parameters.

### 2.5.1. Seahorse XF Cell Energy Phenotype Test

For this test, 20000 cells were seeded in a 96 well Seahorse tissue culture plate (Agilent Technologies). These cells were then left overnight to adhere in a 37°C CO_2_ incubator. A sensor cartridge was placed in XF Calibrant fluid and also left to incubate overnight at 37°C in a non-CO_2_ incubator. The following day, 1mM pyruvate, 2mM glutamine and 10mM glucose were supplemented to Seahorse XF Base medium to make assay medium. This prepared assay medium was pH adjusted to 7.4 and kept at 37°C until needed. The cell culture growth medium from the 96 well tissue culture plate was thereafter removed, and the cells were washed with warmed assay medium, before further addition of 180uL of assay medium to the wells. The tissue culture plate was then placed in a non-CO_2_ incubator at 37°C for an incubation period of 45mins - 1 hour. A stressor mix consisting of a combination of Oligomycin (an inhibitor of ATP synthase) and Carbonyl cyanide-4 (trifluoromethoxy) phenylhydrazone (FCCP) (a mitochondrial uncoupling agent), was made up for addition to the tissue culture plate through injection ports on the previously incubated sensor cartridge during the assay. The loaded sensor cartridge was then placed over the tissue culture plate and the assay was consequently run for 2hrs as per the manufacturer’s given instructions.

### 2.5.2. Seahorse XF Glycolysis Stress Test

For this test, 20000 cells were seeded in a 96 well Seahorse tissue culture plate (Agilent Technologies). These cells were then left overnight to adhere in a 37°C CO_2_ incubator. A sensor cartridge was also left to incubate overnight at 37°C in a non-CO_2_ incubator. The following day, assay medium was first prepared by supplementing Seahorse XF Base medium with 1mM glutamine. This prepared assay medium was pH adjusted to 7.4 and kept at 37°C until the assay was run. The cell culture growth medium from the 96 well tissue culture plate was then removed, and the cells washed with warmed assay medium, before further addition of 180uL of assay medium. The tissue culture plate was then placed in a non-CO_2_ incubator at 37°C for an incubation period of 1 hour. The glycolysis stress test reagents, Oligomycin (ATP synthase inhibitor), Glucose and 2-deoxy-glucose (2-DG, which is a competitive inhibitor of glucose), were prepared for addition to the tissue culture plate during the assay through individual injection ports on the previously incubated sensor cartridge. The loaded sensor cartridge was then placed over the tissue culture plate and the assay was consequently run for 2hrs according to the manufacturer’s instructions. The test reagents were injected in a sequential order during the assay; Firstly, Oligomycin, followed by Glucose, and finally the 2-DG.

### 2.5.3. Seahorse XF Cell Mito Stress Test

For this test, 20000 cells were seeded in a 96 well Seahorse tissue culture plate (Agilent Technologies). These cells were then left overnight to adhere in a 37°C CO_2_ incubator. A sensor cartridge was placed in XF Calibrant fluid and also left to incubate overnight at 37°C in a non-CO_2_ incubator. The following day, with 100mM pyruvate, 200mM glutamine and 2.5M glucose were supplemented to Seahorse XF Base medium to make assay medium. This prepared assay medium was pH adjusted to 7.4 and kept at 37°C until the assay was run. The cell culture growth medium from the 96 well tissue culture plate was then removed, and the cells washed with warmed assay medium, before further addition of 180uL of assay medium. The tissue culture plate was then placed in a non-CO_2_ incubator at 37°C for an incubation period of 1 hour. Three stressor compounds were made up for addition to the tissue culture plate through individual injection ports on the previously incubated sensor cartridge during the assay. The stressors were FCCP (a mitochondrial uncoupling agent), Oligomycin (an inhibitor of ATP synthase) and a combination of Rotenone + antimycin A (inhibitors of complex I and III respectively of the ETC). The loaded sensor cartridge was then placed over the tissue culture plate and the assay was consequently run for 2hrs as per the instructions from the manufacturer. The stressors were injected in a sequential order during the assay; Firstly, FCCP, followed by Oligomycin, and finally the combination of Rotenone + antimycin A.

1. **Statistical Analysis**

Graphpad Prism (Version 6, Graphpad Software Inc.) was utilized for analysis of the raw data with results being expressed as means ± SEM. A One-way or two-way ANOVA with Tukey HSD or Bonferroni’s post-hoc test was used for the inter-group comparison of data sets. P-values less than: 0.05; 0.01 and 0.001 indicated statistically significant different values. Results are from data of at least 3 independent experiments each performed in triplicate or otherwise stated in figure legends.
